# The Utility of Indocyanine Green Angiography in Breast Reconstruction to Detect Mastectomy Skin Flap Necrosis and Free Flap Perfusion: An Umbrella Review

**DOI:** 10.3390/bioengineering11101025

**Published:** 2024-10-15

**Authors:** Nicholas Fadell, Flora Laurent, Sai Anusha Sanka, Esther Ochoa, Lauren Yaeger, Xiaowei Li, Matthew D. Wood, Justin M. Sacks, Saif Badran

**Affiliations:** Division of Plastic and Reconstructive Surgery, Department of Surgery, Washington University School of Medicine, St. Louis, MO 63110, USA; f.nick@wustl.edu (N.F.); flaurent@wustl.edu (F.L.); a.sanka@wustl.edu (S.A.S.); eochoa@wustl.edu (E.O.); yaegerl@wustl.edu (L.Y.); xiaoweili@wustl.edu (X.L.); woodmd@wustl.edu (M.D.W.)

**Keywords:** indocyanine green angiography, breast reconstruction, mastectomy skin flap necrosis, breast fat necrosis

## Abstract

Two of the greatest challenges in breast reconstruction are mastectomy skin flap necrosis (MSFN) and autologous flap failure. This review summarizes current evidence regarding the usage of indocyanine green angiography (ICGA) in breast reconstruction, identifies knowledge gaps, and provides directions for future studies. An umbrella review was conducted to identify related syntheses in Embase, Ovid Medline, Scopus, the Cochrane Central Register of Controlled Trials, the Cochrane Database of Systematic Reviews, and the Clinical Trials databases. Data were extracted from systematic reviews (SRs) and meta-analyses (MAs) that discussed the use of ICGA in breast reconstruction. Sixteen syntheses were included (10 SRs and 6 MAs). Syntheses showed much evidence that ICGA usage typically reduces MSFN rates. However, it tends to overpredict necrosis and is best utilized in high-risk patients or those with an unclear clinical picture. ICGA is also useful in autologous breast reconstruction by reducing rates of breast fat necrosis (BFN), total flap loss, and reoperation. ICGA usage may also aid in perforator mapping and selection intraoperatively, with minimal complication risk. Most syntheses had moderate quality scores; however, they were small with significant heterogeneity in protocols and complication definitions. The use of ICGA in breast reconstruction is safe and useful in decreasing rates of MSFN, BFN, and reoperation after free flap reconstruction.

## 1. Introduction

Breast cancer is the most common cancer among women worldwide, making up 25% of all cancer cases [1,2]. As treatment and survival rates improve, additional women seek breast reconstruction post mastectomy [3]. One of the challenges faced by both general and plastic surgeons during the mastectomy and reconstruction period is complications related to poor tissue perfusion, mainly mastectomy skin flap necrosis (MSFN) and breast fat necrosis (BFN) after autologous reconstruction [4]. These have been reported in up to 30% and 11.3% of cases, respectively [5,6], and can significantly affect patient outcomes and potentially delay their oncological treatment.

The major surgical challenge when attempting to prevent MSFN remains to find the ideal superficial plane of dissection between subcutaneous fat and breast tissue, which is indistinct even microscopically in most cases [7]. Additional factors include patient-related factors such as higher body weight, smoking history, and previous radiation, as well as surgical factors, including the type of mastectomy and skin incision design [8]. Ultimately, MSFN can result in immediate or delayed wound healing problems and a higher risk of infection, which can have a significant impact on patient outcomes. On the other hand, BFN represents a more subtle problem that occurs due to adipocyte hypoperfusion, resulting in non-suppurative inflammation and cell death [6]. This results in cosmetically undesirable palpable nodules in the breast tissue, which can also be potentially suspicious for malignancy recurrence.

Several methods have been examined to accurately assess tissue perfusion during and after mastectomy and autologous breast reconstruction surgeries to accurately and promptly predict hypoperfusion-related complications. While clinical observation remains the traditional gold-standard method for intraoperative perfusion assessment, this can be unclear and misleading in certain cases due to several factors, including tissue edema, low blood pressure, and lack of surgeon experience [9]. Thus, additional adjunct modalities for objectively measuring intraoperative tissue perfusion have surfaced, such as indocyanine green angiography (ICGA) and fluorescein angiography (FA) with Wood’s lamp.

Indocyanine green (ICG) is an iodide dye that can be injected intravenously. It binds to plasma proteins and fluoresces when laser-excited between 705 and 805 nm, and this fluorescence can be captured in real time using a camera [10]. Intraoperatively, it has been used in various surgical fields to capture areas of poor tissue perfusion, as the level of fluorescence decreases with decreasing perfusion [11]. ICGA functions similarly to fluorescein dye angiography, in which fluorescein accumulates in the extracellular space and emits fluorescence in response to ultraviolet light [5]. ICGA is advantageous due to deep penetration and low leakage, which contribute to its superiority over fluorescein angiography due to the latter’s tendency to overestimate ischemia and longer half-life, which makes multiple evaluations within a single operation impractical [4].

In mastectomy and breast reconstruction surgeries, ICGA has been used to examine tissue perfusion both quantitatively and qualitatively [12]. An advantage of this technique is the identification of areas of hypoperfusion that require debridement and would otherwise be missed on clinical examination. However, drawbacks of ICGA include the tendency to overpredict ischemia as well as the potential for inter-observer variability, as no consensus yet exists for objectively assessing tissue viability [13].

Several studies have looked at the utility of ICGA in predicting MSFN and BFN after autologous reconstruction [4,14,15,16,17,18,19,20,21,22,23,24,25,26,27,28,29]. These have been summarized in several systemic reviews and meta-analyses [5,10,13,30,31,32,33,34,35,36,37,38,39,40]. However, these studies lack consistency regarding study population and usage protocols, including ICG dosage and timing [36,38]. Another area of heterogeneity includes reported outcome measures [14,15,23,25,28,29]. The purpose of this umbrella review is to summarize these syntheses regarding the usage of ICGA in mastectomy and autologous breast reconstruction, as well as provide insights and directions for future studies.

## 2. Materials and Methods

### 2.1. Search Strategy

A literature search for records including the concepts of mastectomy and ICG angiography was carried out with the help of a specialized medical librarian (LHY). The search strategy was created using a combination of keywords and controlled vocabulary in Embase.com 1947-, Ovid Medline 1946-, Scopus 1823-, the Cochrane Central Register of Controlled Trials (CENTRAL), the Cochrane Database of Systematic Reviews (CDSR), and Clinicaltrials.gov 1997-. All search strategies were completed on 19 October 2023, with no added limits. A total of 2772 results were found; 1030 duplicate records were deleted using Covidence.org, resulting in a total of 1742 unique citations included in the project library. Fully reproducible search strategies for each database can be found in Appendix A.

### 2.2. Inclusion and Exclusion Criteria

Articles were included if they were systematic reviews or meta-analyses that synthesized data regarding the use of ICGA during mastectomy and/or breast reconstruction. Non-synthesis papers were excluded, as well as lone abstracts and non-English studies.

### 2.3. Article Screening and Data Extraction

Abstracts were screened separately by two reviewers (NF and FL), with discrepancies resolved by the senior author (SB). Full-text screening and data extraction were also performed in duplicate. For each article, the study type was recorded, as well as the patient population and types of procedures examined. Data were categorized into findings related to the use of the ICGA in predicting MSFN and BFN after mastectomy and autologous breast reconstruction.

### 2.4. Quality Assessment

A MeaSurement Tool to Assess Systematic Reviews-2 (AMSTAR-2) was used to assess the quality of included syntheses [41]. This is a 16-safeguard tool that allows methodological measurement of the quality of systematic reviews and meta-analyses.

## 3. Results

### 3.1. Study Selection and Quality Assessment

**Figure 1:**
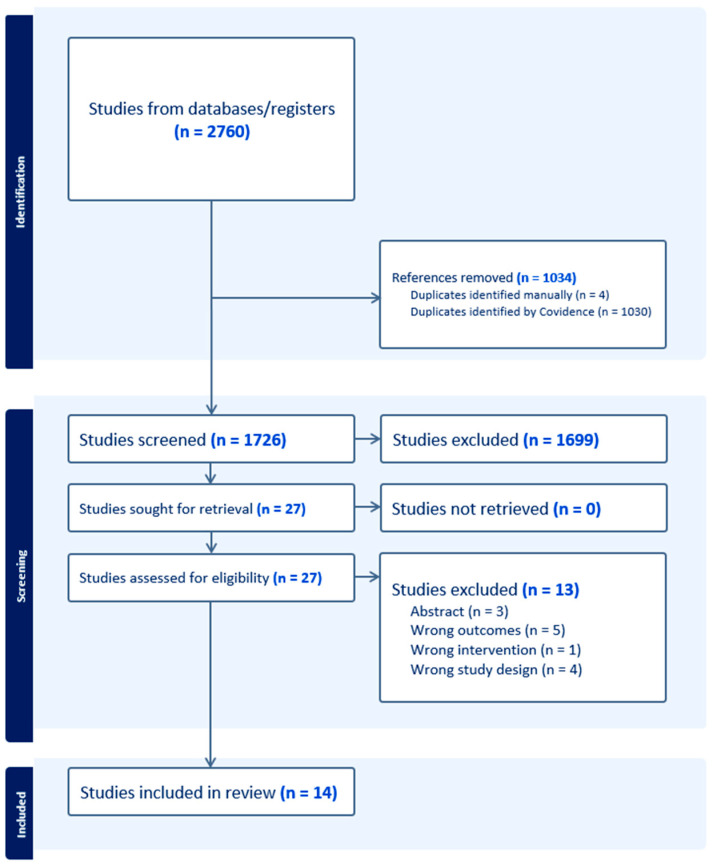
PRISMA flow diagram.

The search strategy resulted in 1726 studies after excluding 1034 duplicates. These studies were screened by title and abstract for eligibility, and 1699 studies were excluded for irrelevancy. The remaining 27 studies were screened by full text. Four studies were excluded due to being the wrong design, three were excluded for being abstracts without a full text, and six were excluded for using ICGA for the wrong intervention. This left a total of 14 syntheses: 8 systematic reviews (SRs) and 6 meta-analyses (MAs). A PRISMA flow diagram is shown in Figure 1.

**Figure 2:**
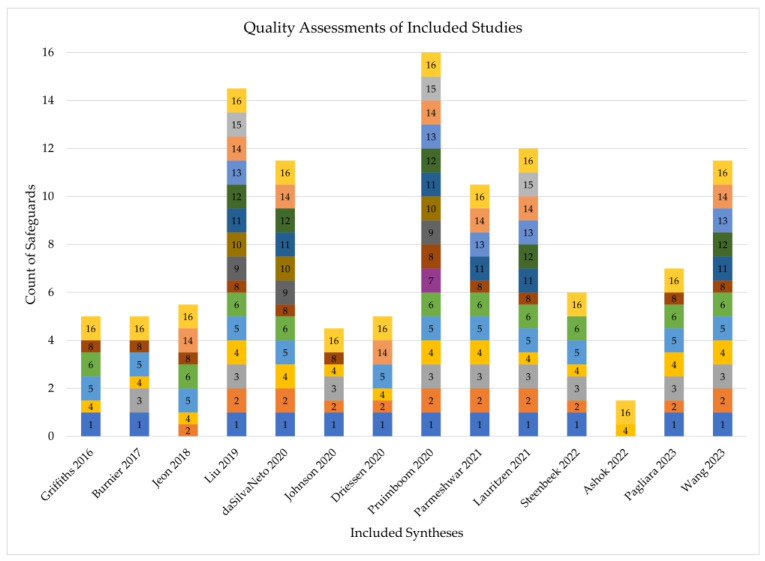
AMSTAR-2 scores of included syntheses [5,10,13,30,31,32,33,34,35,36,37,38,39,40]. Each numbered section represents an AMSTAR-2 safeguard. A thick box indicates the synthesis contained the safeguard, a thin box indicates it was partially present, and missing numbers indicate the safeguard was not present.

The earliest review was conducted in 2017, and the latest in 2023. Thirteen studies reported outcome data on the use of ICGA in predicting MSFN, while seven studies reported on the usage of ICGA in predicting necrosis after autologous breast reconstruction. AMSTAR-2 scores are displayed in Figure 2. Quality assessment revealed that most syntheses were of moderate quality, with an average score of 8.3/16 and scores ranging from 1.5/16 to 16/16. The most common reasons for score deduction were failing to provide a list of excluded studies with reasoning and failing to provide sources of funding for included studies.

### 3.2. The Utility of ICGA in Predicting MSFN

A total of eight SRs and five MAs that discussed the use of ICGA for preventing MSFN were identified (Table 1). Four meta-analyses reported a statistically significant reduction in incidence of MSFN with the use of ICGA [10,36,38,39] and one found no statistical benefit [34]. The greatest benefit reported was an odds ratio of 0.54 (95% CI 0.38–0.77) for MSFN development after mastectomy and reconstruction with intraoperative ICGA versus with clinical judgment alone [10]. Within systematic reviews examining the topic, a wide range of conclusions have been reported in the existing literature. The sensitivity and specificity of ICGA for detecting MSFN have ranged from 38 to 100% and 68 to 91%, respectively [5]. Reductions in MSFN rates after the use of ICGA have been reported as high as 84% [27], while other articles have found no statistical benefit [30]. Notably, ICGA has been found to possibly overpredict areas of skin flap necrosis by as high as 72% [23]. Syntheses recommend the use of ICGA in high-risk patients, such as those with high BMI and smokers, as well as when clinical evaluation of skin flaps is equivocal [37,40].

Based on these syntheses, ICGA has the potential to be useful for predicting areas of MSFN with high sensitivity. However, current conclusions are varied, and surgeons should be aware of the tendency for ICGA to overpredict areas of necrosis. It is challenging to draw definitive conclusions from the existing literature due to heterogeneity. Individual studies have differed in types of reconstructions included, ICG dosage and timing of administration, and classification of postoperative skin flap necrosis. More specifically, some classified necrosis by required intervention [14,15,26], others classified necrosis by affected/exposed tissue [23,25,28,29], and some did not explicitly state how necrosis was classified and what degree was included [16,22]. This makes it difficult to define and quantify the effect of ICGA on this outcome, as certain severities of necrosis included in one study may have been excluded in another. Further prospective studies with large, multi-institutional cohorts, standardized ICG protocols, and consistent necrosis definitions are needed to definitively quantify the benefits of ICGA for predicting MSFN.

### 3.3. The Utility of ICGA in Predicting BFN

Four SRs and four MAs examined the effect of ICGA on the incidence of fat necrosis in this review (Table 2). Of the four meta-analyses, three reported a significant reduction in incidence of fat necrosis with the use of ICGA [33,34,39] and one did not include fat necrosis as an outcome in pooled analysis [36]. The greatest reported benefit of ICGA when used for the prediction of breast flap necrosis was an odds ratio of 0.31 (*p* = 0.006) [33]. This benefit is likely due to the ability of ICGA to identify poorly perfused flap tissue intraoperatively and guide under perfused tissue resection [32]. No meta-analyses reported a statistically significant reduction in total flap loss with the use of ICGA, and one reported that ICGA reduced the risk of both major complications requiring operative intervention and minor complications managed conservatively (OR 0.62 and OR 0.53, respectively) [39]. A systematic review of the existing literature showed a wide variety of conclusions, with some studies reporting no statistically significant benefit of ICGA [14,24] and others reporting a nearly 100% reduction in postoperative fat necrosis when intraoperative ICGA was used [31].

Many prior studies and syntheses have reported a statistically significant benefit of ICGA in reducing postoperative breast flap necrosis. However, like MSFN, the generalizability of these conclusions is limited due to varying ICGA protocols, small sample sizes, and heterogenous definitions of fat necrosis. Fat necrosis was sometimes classified by the size of a palpable mass within the breast [17], some was further classified based on severity [18,21], and other studies [19,20] used a classification system based on a review performed in 2013 by Lie et al. [42]. Large, multi-center prospective trials with consistent ICGA protocols, a follow-up time of at least one year, and standardized inclusion and exclusion criteria for diagnoses of breast fat necrosis are needed.

## 4. Discussion

Several studies [4,14,15,16,17,18,19,20,21,22,23,24,25,26,27,28,29] have looked at the usage of ICGA in breast reconstruction. However, these studies have been criticized for their small sample size, retrospective study design, significant heterogeneity [36], short-term follow-up [33], and inconsistent usage protocols [36,38]. This umbrella review summarizes 14 syntheses examining evidence on the benefits of ICGA during mastectomy and breast reconstruction. Overall, ICGA appears promising as a tool to reduce the risk of MSFN and BFN in patients undergoing breast reconstruction as well as reduce the risk of returning to the operating room. It has been shown to reduce rates of MSFN by up to 84% and rates of BFN by up to 69%. However, current evidence for these claims is severely limited by significant heterogeneity in studies’ patient populations, ICGA protocols, and definitions of ischemic complications.

To address the abovementioned challenges, a large, multi-center, prospective randomized control trial should be designed with consistent ICGA doses and protocols. Based on current recommendations in the literature, for such a trial we recommend an intravenous ICG dose of 2.5 mg/mL followed by a 10 mL normal saline flush 90 s prior to perfusion assessment, a working distance of 20 cm from the skin, turning off all room lights during assessment, and the avoidance of epinephrine-containing tumescent solution for study patients [43]. Mastectomy skin flap necrosis should be grouped into necrosis requiring debridement or necrosis managed non-surgically, and breast flap fat necrosis should be stratified by palpability, visibility, and presence of symptoms [44]. Follow-up for each patient should be at least one year to adequately capture the incidence of fat necrosis.

Another important focus point for future studies would be the standardization of outcome measures. There is a wide variety of methods for identification and stratification of MSFN and fat necrosis, and this makes it difficult to compare and generalize the findings of different studies examining these outcomes [33,39]. While classifications of MSFN exist, a consensus on a standardized definition has yet to be reached [45]. Reaching such a consensus would be an important step in the ability to compare the outcomes of separate studies examining outcomes of ICGA on breast reconstruction.

While the use of ICGA shows great promise in improving outcomes of breast reconstruction, other modalities for measuring perfusion are also being studied for this purpose. One such modality is hyperspectral imaging (HSI), in which tissue is illuminated with a broadband light source and a hyperspectral camera captures the reflection of a large number of wavelengths across the electromagnetic spectrum [46]. This data can then be used to provide information on oxyhemoglobin and deoxyhemoglobin levels, tissue oxygenation, and near-infrared perfusion [47]. HSI is non-invasive, radiation-free, and requires no dye or medication. Recent research has shown that data from HSI on tissue oxygenation and near-infrared perfusion have correlated to areas of necrosis of both mastectomy skin flaps and free flaps [48]. Additionally, HSI has been used in conjunction with artificial intelligence and deep learning models to assist with achieving negative margins in breast-conserving surgery, as AI excels at the interpretation of the large volume of data HSI can generate [46]. However, current applications of HSI technology are physically large and expensive, making them difficult to integrate into surgical workflows [49]. Future work will need to focus on making HSI faster, more portable, and more accessible, as well as the standardization of protocols and image interpretation.

Another promising modality for the prediction of MSFN and BFN is laser speckle contrast imaging (LSCI). In this technique, tissue is illuminated with 785 nm laser light, and a camera is used to analyze the scatter, or speckle, pattern to quantify perfusion using “perfusion units” (PU) [50]. LSCI can be performed in only a few seconds, requires no contrast dye, and is cheaper per case than ICGA [51]. It has been successfully applied in animal studies of free flap perfusion to predict flap necrosis, and its perfusion measurements have also been shown to correlate with those from ICGA [52,53]. In human studies, LSCI has been able to display reductions in DIEP flap perfusion that correlate with Holm classifications, as well as predict areas of DIEP flap necrosis based on a 30 PU threshold [51]. While quite promising, LSCI suffers from similar drawbacks as ICGA in that its application and interpretation must still be standardized to truly understand its benefit. LSCI data are also quite sensitive to motion artifacts and can only display perfusion data to a depth of 300 μm, meaning it may miss deeper fat necrosis [51].

Lastly, photoacoustic imaging (PAI) is a modality that has seen use in many other medical disciplines that may prove useful in preventing MSFN and BFN. PAI is a hybrid imaging modality that uses nanosecond pulses of laser light that heat tissues, which generate acoustic waves via thermoelastic expansion that are detected using ultrasound [54]. Due to their differences in intrinsic optical absorption, PAI can differentiate between deoxyhemoglobin and hemoglobin to measure oxygenation and perfusion. It can be used with or without contrast dye, such as ICG, and is effective up to a depth of several centimeters. In a mouse model, PAI has been shown to be able to map perforators for flap harvest and identify reduced perfusion in areas that go on to develop necrosis [55]. However, PAI technology must become much more portable and requires much more clinical testing in breast reconstruction to become standard for improving outcomes. Ultimately, there is a large volume of research being conducted to develop a cheap, safe, and reliable imaging modality that allows surgeons to identify and excise tissue at high risk of necrosis in breast reconstruction. With each of these techniques, improvements in artificial intelligence and deep learning models may allow for an even greater predictive accuracy and account for patient factors such as BMI, smoking, and medical comorbidities.

## 5. Conclusions

The use of ICGA in mastectomy and breast reconstruction is useful in reducing perfusion-related complications such as mastectomy skin flap necrosis, fat necrosis, and partial flap loss. Further studies are needed to identify an optimal standardized ICGA protocol and outcome measures.

## Figures and Tables

**Table 1:** Syntheses that report incidence of MSFN with and without ICGA.

Study ID	Article Type	Patient Population	Article Date Range	Included Studies	Duration of Follow-Up	Main Findings	Secondary Findings	Remaining Evidence Gaps
Wang et al., 2023 [34]	SR and MA	1814 patients who underwent DIEP flap breast reconstruction with and without ICGA	2006–2022	5 prospective noncomparative, 5 retrospective noncomparative, 2 prospective comparative, 9 retrospective comparative, 1 RCT	Over 6 months	ICGA did not significantly alter the risk of mastectomy skin flap necrosis in DIEP flap reconstructions (RR 0.9, *p* = 0.88).	ICGA did not significantly alter the risk of seroma (RR 1.21, *p* = 0.68), hematoma (RR 0.94, *p* = 0.71), dehiscence (RR 1.71, *p* = 0.18), and infection (RR 0.77, *p* = 0.51)	Most included studies were retrospective and the MA only included five studies, limiting generalizability and power for detecting differences in each outcome measure. There was also significant heterogeneity among included studies regarding follow-up, ICGA protocols, and criteria for including versus excluding degrees of necrosis.
Pagliara et al., 2023 [37]	SR	1151 breasts undergoing mastectomy with immediate, implant-based reconstruction	Not specified	1 descriptive, 1 systematic review, 6 prospective, 6 retrospective	Not specified	Unclear effect of ICGA alone on MSFN, but according to one study ICGA + SPY analysis may significantly reduce risk. ICGA may increase precision for tissue debridement during mastectomy and reconstruction. Authors recommend using ICGA intraoperatively when clinical assessment is uncertain.	Widespread ICGA + SPY may not be cost effective, but is advantageous when used on high-risk patients such as smokers and obese patients	This review is limited by significant heterogeneity in the protocols by which skin flaps were assessed as well as how necrosis was classified and how comorbidities were factored into results.
Ashok et al., 2022 [32]	SR	All breast surgeries with any use of ICG	1977–2021	55 included, breakdown not specified	Not specified	For MSFN, ICGA is superior to clinical assessment when predicting necrosis. One study reported a 90% sensitivity and a 100% specificity.	None	The review presents data on several studies regarding ICGA and preventing MSFN but provides no pooled analysis and does not discuss any heterogeneity in studies or studies with alternative conclusions.
Steenbeek et al., 2022 [35]	SR	Free flap breast reconstructions with perforator imaging modalities	2004–2020	168 studies, breakdown not specified	Not specified	ICGA can be used to assist in early identification of MSFN to prevent complications.	ICGA can be used to support decisions for which perforators to use for free flap reconstruction. ICGA is more precise than CTA for perforator mapping but can only find superficial vessels.	This review touches on perfusion assessment of skin flaps but mainly focuses on perforator assessment in free flap surgery.
Lauritzen, Damsgaard, 2021 [39]	SR and MA	4314 breast reconstructions with ICGA, mean age 49.6	2010–2020	1 RCT study, 2 clinical trials, 4 comparative studies, 8 cohort studies, and 11 case controls	2 weeks to 24 months, reported in 23 of 26 papers	In pooled analysis when evaluating mastectomy flaps with implant reconstruction, ICGA significantly reduced major complications requiring return to OR (OR 0.56, *p* = 0.001) and loss of reconstruction (OR 0.46, *p* = 0.006). When evaluating mastectomy skin flaps with autologous reconstruction, ICGA reduced risk of minor complications (OR 0.41, *p* = 0.0001) but had no significant effect on major complications or losses of reconstruction. ICGA correlates significantly with reduced risk of major complications and reconstruction loss in mastectomy skin flaps.	None	The studies included in this synthesis are heterogenous in their design and definition of fat necrosis. The majority of these were retrospective in nature and may be subject to biases or confounders.
Pruimboom et al., 2020 [36]	SR and MA	1589 women undergoing 2199 SSMs with immediate autologous or prosthetic reconstruction	2014–2018, searched 1946–2019	7 nonrandomized, retrospective cohort studies and 2 nonrandomized, prospective cohort studies	90 days to 6 months	Authors rated quality of evidence very low for most outcomes. For postoperative MSFN per patient, ICGA had an uncertain effect on risk (RR 0.79, 95%CI 0.40 to 1.56). For postoperative MSFN per breast, ICGA may reduce risk (RR 0.62, 95% CI 0.48 to 0.82). Overall, ICGA may reduce incidence of some complications, but specifics are unclear.	ICGA reduced reoperation when examined per patient (RR 0.5, 95% CI 0.35–0.72) and per breast (RR 0.65, 95% CI 0.47–0.92) but very low quality of evidence. When examining infection risk per breast, ICGA reduced risk (RR 0.65, 95% CI 0.44 to 0.97) but had low evidence quality. When examining hematoma per patient, ICGA reduced risk (RR 0.87, 95% CI 0.3–2.53) although had low evidence quality. No clear effect on risk for dehiscence.	Most of the available included studies are retrospective, which may introduce confounding variables, and the low sample sizes and heterogeneity of included studies make the quality of evidence low for pooled analysis.
Driessen et al., 2020 [30]	SR	1656 patients undergoing mastectomy with or without immediate implant reconstruction	2005–2018	nonrandomized cohort studies, 6 retrospective, 10 prospective	Not specified	When using ICGA and SPY, absolute perfusion cutoffs of 7–8 were reported for tissue debridement. For relative perfusion, suggested cutoffs are 34% and 25–33%. Significant decrease in MSFN with ICG, but there is no consensus on which parameters to use for SPY. ICGA tends to increase surgical debridement intraoperatively.	None	Heterogeneity of included studies made pooled analysis impossible, limiting the conclusions that can be drawn. Small sample sizes in included studies and limited follow-up also limit generalizability.
Johnson et al., 2020 [40]	SR	ICGA in mastectomy skin flap assessment: 1005 patients ICGA in NAC perfusion assessment: 79 patients + 149 NACs ICGA in implant-based reconstruction: 604 patients ICGA in autologous reconstruction: 1595 patients	Not specified	43 total studies, breakdown not provided	Not specified	In mastectomy, ICGA reduces risk of ischemic complications but is most important when clinical evaluation is unclear. For mastectomy with implant-based reconstruction, ICGA can be used to determine skin flap and NAC perfusion to aid in adjusting implant volume and debriding tissue.	None	Some confounders, such as smoking and epinephrine-containing tumescent solution, were not adjusted for in included studies and this can confound evidence.
daSilvaNeto et al., 2020 [10]	SR and MA	1948 SSMs or NSM patients with reconstruction, age 26–75	all articles before 30 August 2019	Two studies were cohort, two were case-control, and five were retrospective reviews	90 days to 6 months	For MSFN, clinical assessment alone resulted in significantly higher risk of necrosis than clinical assessment plus ICGA (OR 1.85, *p* = 0.0006). Clinical assessment alone also had significantly higher risk of reoperation (OR 2.05, *p* = 0.0002). Overall, ICGA appears to reduce rates of MSFN and nipple necrosis.	No significant difference in risk between using clinical assessment alone or clinical assessment with ICGA for infection (OR 1.78, *p* = 0.05) or seroma (OR 1.06, *p* = 0.85).	Larger, prospective studies are needed with standardized definitions for necrotic complications and what constitutes a perfusion-related reoperation.
Liu et al., 2019 [38]	SR and MA	902 skin or nipple sparing mastectomies with reconstruction, mean age of four included studies 47–53	searched 1 January 1960 to 1 March 2018	6 retrospective, 1 prospective	3 to 16.9 months	ICGA significantly lowered rates of skin necrosis (9.8% vs. 15.2%, *p* = 0.001). In weighted meta-analysis, ICGA significantly reduced rate of MSFN (OR 0.56, 95% CI 0.35–0.89, *p* = 0.02, I^2^ = 40%).	ICGA significantly lowered rates of complication (26.9% vs 30.7%, *p* = 0.03) and reoperation (6.8% vs 15.2%, *p* < 0.01). In weighted meta-analysis, ICG significantly reduced rate of reoperation (OR 0.32, 95% CI 0.21–0.49, *p* < 0.00001, I^2^ = 19%) and complications (OR 0.62, 95% CI 0.41–0.94, *p* = 0.06, I^2^ = 54%).	Studies included were largely retrospective with small sample sizes. Did not include cases of necrosis requiring surgery from one study.
Jeon et al., 2018 [5]	SR	2650 breasts undergoing NSM or SSM with immediate reconstruction	searched 1946–2017	8 prospective cohort, 4 retrospective case series, 3 prospective case series, 1 retrospective case-control study, 1 prospective pilot trial, 1 cost analysis study	Not specified	ICGA decreased rates of MSFN and reoperation in mastectomy with immediate reconstruction. ICGA tends to overpredict necrosis and underperfusion. Many variables also affect accuracy of ICGA, including room temperature, blood pressure, systemic vascular resistance, and ambient light.	None	Significant heterogeneity exists in the included studies’ definitions of skin flap necrosis.
Burnier et al., 2017 [31]	SR	4368 plastic surgery patients	2002–2015	41 including RCTs, feasibility studies, non-randomized comparative studies, technique descriptions, retrospective reviews	Not specified	Intraoperative ICGA can predict mastectomy skin flap necrosis accurately during breast reconstruction. ICGA has been used to lower incidence of mastectomy skin necrosis and unexpected perfusion-related reoperations	None	Heterogeneity among included studies limits conclusions that can be drawn, and low follow-up times in some included studies means all instances of breast flap necrosis may not have been captured.
Griffiths et al., 2016 [13]	SR	Any breast reconstruction using ICGA	2004–2014	9 retrospective studies and 4 prospective studies	Not specified	Current evidence suggests use of ICGA reduces incidence of MSFN. One study found a 95% correlation between findings on ICGA and subsequent mastectomy skin flap necrosis, with a sensitivity of 100% and a specificity of 91%. However, absolute perfusion via SPY-Q software is prone to being confounded by variables such as smoking and the use of epinephrine-containing tumescent solution.	None	Data presented are qualitative, with no pooled analysis and no discussion of heterogeneity or confounders.

MSFN: Mastectomy skin flap necrosis; ICGA: Indocyanine green angiography; ICG: Indocyanine green; SR: Systematic review; MA: Meta-analysis; DIEP: Deep inferior epigastric artery perforator; RR: Relative risk; CTA: Computed tomography angiography; RCT: Randomized control trial; OR: Odds ratio; SSM: Skin sparing mastectomy; CI: Confidence interval; NAC: Nipple areolar complex; NSM: Nipple sparing mastectomy.

**Table 2:** Syntheses that report incidence of BFN with and without ICGA.

Study ID	Article Type	Patient Population	Article Date Range	Included Studies	Duration of Follow-Up	Main Findings	Secondary Findings	Remaining Evidence Gaps
Wang et al., 2023 [34]	SR and MA	1814 patients who underwent DIEP flap breast reconstruction with and without ICGA	2006–2022	5 prospective noncomparative, 5 retrospective noncomparative, 2 prospective comparative, 9 retrospective comparative, 1 RCT	Not specified	Authors concluded intraoperative ICGA was associated with significantly lower rates of postoperative fat necrosis (RR 0.47, *p* = 0.004) and ischemia-related reoperations (RR 0.41, *p* = 0.03). No significant difference observed for total flap loss (RR 0.89, *p* = 0.76) and partial flap loss (RR 0.25, *p* = 0.09).	ICGA did not significantly alter the risk of seroma (RR 1.21, *p* = 0.68), hematoma (RR 0.94, *p* = 0.71), dehiscence (RR 1.71, *p* = 0.18), and infection (RR 0.77, *p* = 0.51). ICGA may not be cost effective when performed repetitively.	Most included studies were retrospective and the MA only included five studies, limiting generalizability and power for detecting differences in each outcome measure. There was also significant heterogeneity among included studies regarding follow-up, ICGA protocols, and criteria for including versus excluding degrees of fat necrosis.
Ashok et al., 2022 [32]	SR	All breast surgeries with any use of ICG	1977–2021	495 articles, 55 included in results	Not specified	ICGA is very reliable for detecting free flap hypoperfusion and guiding tissue debridement. ICGA frequently does not correlate with clinical signs of hypoperfusion, in which case tissue resection can be delayed while perfusion stabilizes. ICGA has been shown to improve partial flap survival.	None	The review mainly cites evidence from studies on free flaps in general rather than those focusing on breast reconstruction when discussing ICGA and fat necrosis. No pooled analysis or statistical data are presented.
Lauritzen, Damsgaard, 2021 [39]	SR and MA	4314 breast reconstructions with ICGA, mean age 49.6	2010–2020	1 RCT study, 2 clinical trials, 4 comparative studies, 8 cohort studies, and 11 case controls	2 weeks to 24 months, reported in 23 of 26 papers	When evaluating autologous reconstruction, ICGA significantly reduced risk of major complications (OR 0.62, *p* = 0.001) and minor complications (OR 0.53, *p* = 0.028), but had no significant effect on flap loss. ICGA lowers overall complication risk in autologous breast reconstruction	None	The studies included in this synthesis are heterogenous in their design and definition of fat necrosis. The majority of these were retrospective in nature and may be subject to biases or confounders.
Parmeshwar et al., 2021 [33]	SR and MA	355 patients, 824 autologous breast reconstructions, half immediate	1 January 2000 to 1 March 2020	1 prospective randomized trial, 8 retrospective cohort studies	3 months to 1 year	ICG in single-institution studies has been shown to be useful in minimizing flap failure, MSFN, and fat necrosis. ICGA helpful for intraoperative debridement, which reduces necrosis/flap loss. ICGA significantly reduced fat necrosis in autologous flaps (OR = 0.31, *p* = 0.0006) but showed no statistically significant difference in total or partial flap loss.	None	The studies included in this MA had a maximum follow-up time of one year, which may be insufficient to truly determine fat necrosis rates. Studies also used differing definitions of necrosis and some did not specify theirs.
Pruimboom et al., 2020 [36]	SR and MA	1589 women undergoing 2199 SSMs with immediate autologous or prosthetic reconstruction	2014–2018, searched 1946–2019	7 nonrandomized, retrospective cohort studies and 2 nonrandomized, prospective cohort studies	90 days to 6 months	Authors rated the quality of evidence very low for most outcomes and found no clear effect of ICGA on autologous flap necrosis. Overall, ICGA may reduce incidence of some complications, but specifics are unclear.	ICGA reduced reoperation when examined per patient (RR 0.5, 95% CI 0.35–0.72) and per breast (RR 0.65, 95% CI 0.47–0.92) but very low quality of evidence. When examining infection risk per breast, ICGA reduced risk (RR 0.65, 95% CI 0.44 to 0.97) but evidence was of low quality. When examining hematoma per patient, ICGA reduced risk (RR 0.87, 95% CI 0.3–2.53) but still evidence was low quality. ICGA had no clear effect on dehiscence.	Most of the available included studies are retrospective, which may introduce confounding variables. Only two included studies discussed ICGA and breast fat necrosis, so no pooled statistical analysis was conducted.
Johnson et al., 2020 [40]	SR	ICGA in mastectomy skin flap assessment: 1005 patients ICGA in NAC perfusion assessment: 79 patients + 149 NACs ICGA in implant-based reconstruction: 604 patients ICGA in autologous reconstruction: 1595 patients	Not specified	43 studies	Not specified	In autologous reconstruction, ICGA is useful to visualize flap perfusion and is reliable, can decrease fat necrosis rates postoperatively, and can possibly lead to higher BREAST-Q scores. ICGA can reduce rates of unexpected reoperations; however, it tends to overestimate underperfusion and venous congestion.	None	Studies cited in this review have significant heterogeneity in their ICGA protocols and some do not adjust for confounding intraoperative factors, such as mean arterial pressure or epinephrine-containing tumescent solution.
Burnier et al., 2017 [31]	SR	4368 plastic surgery patients	2002–2015	41 including RCTs, feasibility studies, non-randomized comparative studies, technique descriptions, retrospective reviews	Not specified	ICGA can aid in visualizing free flap perfusion, venous congestion, and vasospasm. Not very successful for detecting DIEP perforators but can reduce partial flap loss and flap necrosis.	None	Heterogeneity among included studies limits conclusions that can be drawn, and low follow-up times in some included studies means all instances of breast flap necrosis may not have been captured.
Griffiths et al., 2016 [13]	SR	Any breast reconstruction using ICGA	2004–2014	9 retrospective studies and 4 prospective studies	Not specified	ICGA has demonstrated utility in selection of dominant perforators for free flap reconstruction intraoperatively as well as intraoperative perfusion assessment. Many studies have correlated areas of hypoperfusion identified with ICGA with areas of postoperative flap necrosis. ICGA can also be used to evaluate microanastomotic patency and detect occlusion, thrombosis, and pedicle torsion.	None	Only two studies are mentioned regarding free flap perfusion assessment and breast flap necrosis, and much of the data focus on TRAM flaps where as DIEP flaps are more commonly performed today.

BFN: Breast fat necrosis; ICGA: Indocyanine green angiography; ICG: Indocyanine green; SR: Systematic review; MA: Meta-analysis; DIEP: Deep inferior epigastric artery perforator; RR: Relative risk; RCT: Randomized control trial; OR: Odds ratio; SSM: Skin sparing mastectomy; CI: Confidence interval; NAC: Nipple areolar complex.

## Data Availability

No new data were created or analyzed in this study. Data sharing is not applicable to this article.

## Appendix A. Full Search Strategy

**Embase** 

Date Searched: 19 October 2023

Applied Database Supplied Limits: none

Number of Results: 808

Full Search Strategy:

(‘mastectomy’/exp OR ‘nipple-sparing mastectomy’/exp OR ‘subcutaneous mastectomy’/exp OR ‘skin-sparing mastectomy’/exp OR ‘breast reconstruction’/exp OR (mastectom* OR NACSM OR ‘breast amputation*’ OR ‘breast resection*’ OR mammectom* OR ‘breast reconstruction’ OR mammoplast* OR mastoplast*):ti,ab,kw) AND (‘indocyanine green’/exp OR ‘fluorescence’/de OR ‘fluorescence angiography’/de OR (‘indocyanine green’ OR ‘ICG-based fluorescence’ OR ‘cardio green’ OR ‘cardio-green’ OR cardiogreen OR diagnogreen OR ‘fox green’ OR ICG OR ‘indocyanide green’ OR ‘indocyanin green’ OR ‘spy agent green’ OR ‘tricarbocyanine ii’ OR ujoviridin OR vofaverdin OR wofaferdin OR wofaverdin OR fluorescence OR ‘fluorescent method’ OR ‘fluorescein angiography’ OR ‘fluorescein photography’ OR ‘fluorescence angiophotography’ OR ‘fluorescent angiography’ OR ‘fluorescin angiography’ OR ‘fluoroangiography’ OR ‘fluorescence angiography’):ti,ab,kw)

**Ovid Medline** 

Date Searched: 19 October 2023

Applied Database Supplied Limits: none

Number of Results: 420

Full Search Strategy:

(exp Mastectomy, Subcutaneous/ OR exp Mastectomy/ OR exp Mammaplasty/ OR (mastectom* OR NACSM OR breast amputation* OR breast resection* OR mammectom* OR breast reconstruction OR mammoplast* OR mastoplast*).ti,ab,kf.) AND (exp Indocyanine Green/ OR *Fluorescence/ OR *Fluorescein Angiography/ OR (indocyanine green OR ICG-based fluorescence OR cardio green OR cardio-green OR cardiogreen OR diagnogreen OR fox green OR ICG OR indocyanide green OR indocyanin green OR spy agent green OR tricarbocyanine ii OR ujoviridin OR vofaverdin OR wofaferdin OR wofaverdin OR fluorescence OR fluorescent method OR fluorescein angiography OR fluorescein photography OR fluorescence angiophotography OR fluorescent angiography OR fluorescin angiography OR fluoroangiography OR fluorescence angiography).ti,ab,kf.)

**Scopus** 

Date Searched: 19 October 2023

Applied Database Supplied Limits: none

Number of Results: 1477

Full Search Strategy:

((TITLE-ABS-KEY(mastectom* OR NACSM OR “breast amputation*” OR “breast resection*” OR mammectom* OR “breast reconstruction” OR mammoplast* OR mastoplast*))) AND ((TITLE-ABS-KEY(“indocyanine green” OR “ICG-based fluorescence” OR “cardio green” OR “cardio-green” OR cardiogreen OR diagnogreen OR “fox green” OR ICG OR “indocyanide green” OR “indocyanin green” OR “spy agent green” OR “tricarbocyanine ii” OR ujoviridin OR vofaverdin OR wofaferdin OR wofaverdin OR fluorescence OR “fluorescent method” OR “fluorescein angiography” OR “fluorescein photography” OR “fluorescence angiophotography” OR “fluorescent angiography” OR “fluorescin angiography” OR “fluoroangiography” OR “fluorescence angiography”)))

**The Cochrane Library** 

Date Searched: 19 October 2023

Applied Database Supplied Limits: none

Number of Results

CENTRAL: 54

CDSR: 1

Full Search Strategy:

([mh “Mastectomy, Subcutaneous”] OR [mh “Mastectomy”] OR [mh “Mammaplasty”] OR (mastectom* OR NACSM OR “breast amputation*” OR “breast resection*” OR mammectom* OR “breast reconstruction” OR mammoplast* OR mastoplast*):ti,ab,kw) AND ([mh “Indocyanine Green”] OR [mh “Fluorescence”] OR [mh “Fluorescein Angiography”] OR (“indocyanine green” OR “ICG based fluorescence” OR “cardio green” OR “cardio green” OR cardiogreen OR diagnogreen OR “fox green” OR ICG OR “indocyanide green” OR “indocyanin green” OR “spy agent green” OR “tricarbocyanine ii” OR ujoviridin OR vofaverdin OR wofaferdin OR wofaverdin OR fluorescence OR “fluorescent method” OR “fluorescein angiography” OR “fluorescein photography” OR “fluorescence angiophotography” OR “fluorescent angiography” OR “fluorescin angiography” OR “fluoroangiography” OR “fluorescence angiography”):ti,ab,kw)

**ClinicalTrials.gov** 

Date Searched: 19 October 2023

Number of Results: 12

Full Search Strategy:

mastectomy AND (“Indocyanine Green” OR fluorescence)

## References

[1] Bray F., Ferlay J., Soerjomataram I., Siegel R.L., Torre L.A., Jemal A. (2018). Global cancer statistics 2018: GLOBOCAN estimates of incidence and mortality worldwide for 36 cancers in 185 countries. CA Cancer J. Clin..

[2] Ferlay J., Soerjomataram I., Dikshit R., Eser S., Mathers C., Rebelo M., Parkin D.M., Forman D., Bray F. (2015). Cancer incidence and mortality worldwide: Sources, methods and major patterns in GLOBOCAN 2012. Int. J. Cancer.

[3] Yi M., Kronowitz S.J., Meric-Bernstam F., Feig B.W., Symmans W.F., Lucci A., I Ross M., Babiera G.V., Kuerer H.M., Hunt K.K. (2011). Local, regional, and systemic recurrence rates in patients undergoing skin-sparing mastectomy compared with conventional mastectomy. Cancer.

[4] Komorowska-Timek E., Gurtner G.C. (2010). Intraoperative perfusion mapping with laser-assisted indocyanine green imaging can predict and prevent complications in immediate breast reconstruction. Plast. Reconstr. Surg..

[5] Jeon F.H.K., Varghese J., Griffin M., Butler P.E., Ghosh D., Mosahebi A. (2018). Systematic review of methodologies used to assess mastectomy flap viability. BJS Open.

[6] Khansa I., Momoh A.O., Patel P.P., Nguyen J.T., Miller M.J., Lee B.T. (2013). Fat necrosis in autologous abdomen-based breast reconstruction: A systematic review. Plast. Reconstr. Surg..

[7] Beer G.M., Varga Z., Budi S., Seifert B., Meyer V.E. (2002). Incidence of the superficial fascia and its relevance in skin-sparing mastectomy. Cancer.

[8] Robertson S.A., Jeevaratnam J.A., Agrawal A., Cutress R.I. (2017). Mastectomy skin flap necrosis: Challenges and solutions. Breast Cancer Targets Ther..

[9] Singer R., Lewis C.M., Franklin J.D., Lynch J.B. (1978). Fluorescein Test for Prediction of Flap Viability during Breast Reconstructions. Plast. Reconstr. Surg..

[10] Neto E.d.S., Figueiredo P.H.M., Moro M.G., Assumpção C.B., Perina A.L.F., da Costa F.P.P., Faria E.P., de Oliveira A.C.V., Prates R.A. (2020). Use of laser-assisted indocyanine green angiography in breast reconstruction: Systematic review and meta-analysis. J. Surg. Oncol..

[11] Reinhart M.B., Huntington C.R., Blair L.J., Heniford B.T., Augenstein V.A. (2015). Indocyanine Green. Surg. Innov..

[12] Pruimboom T., Van Kuijk S.M.J., Qiu S.S., van den Bos J., Wieringa F., Van Der Hulst R.R.W.J., Schols R.M. (2020). Optimizing Indocyanine Green Fluorescence Angiography in Reconstructive Flap Surgery: A Systematic Review and Ex Vivo Experiments. Surg. Innov..

[13] Griffiths M., Chae M.P., Rozen W.M. (2016). Indocyanine green-based fluorescent angiography in breast reconstruction. Gland. Surg..

[14] Duggal C.S., Madni T., Losken A. (2014). An Outcome Analysis of Intraoperative Angiography for Postmastectomy Breast Reconstruction. Aesthetic Surg. J..

[15] Diep G.K., Hui J.Y.C., Marmor S., Cunningham B.L., Choudry U., Portschy P.R., Tuttle T.M. (2016). Postmastectomy Reconstruction Outcomes After Intraoperative Evaluation with Indocyanine Green Angiography versus Clinical Assessment. Ann. Surg. Oncol..

[16] Sood M., Glat P. (2013). Potential of the SPY intraoperative perfusion assessment system to reduce ischemic complications in immediate postmastectomy breast reconstruction. Ann. Surg. Innov. Res..

[17] Hembd A.S., Yan J., Zhu H., Haddock N.T., Teotia S.S. (2020). Intraoperative Assessment of DIEP Flap Breast Reconstruction Using Indocyanine Green Angiography: Reduction of Fat Necrosis, Resection Volumes, and Postoperative Surveillance. Plast. Reconstr. Surg..

[18] Malagón-López P., Vilà J., Carrasco-López C., García-Senosiain O., Priego D., Ibañez J.F.J., Higueras-Suñe C. (2019). Intraoperative Indocyanine Green Angiography for Fat Necrosis Reduction in the Deep Inferior Epigastric Perforator (DIEP) Flap. Aesthetic Surg. J..

[19] Michi M., Verduijn P.S., Corion L.U.M., Vahrmeijer A.L., Mulder B.G.S. (2022). Assessment of deep inferior epigastric perforator flap perfusion with near-infrared fluorescence: A pilot study and description of a standardized working protocol. J. Plast. Reconstr. Aesthetic Surg..

[20] Varela R., Casado-Sanchez C., Zarbakhsh S., Diez J., Hernandez-Godoy J., Landin L. (2020). Outcomes of DIEP Flap and Fluorescent Angiography: A Randomized Controlled Clinical Trial. Plast. Reconstr. Surg..

[21] Yoo A., Palines P.A., Mayo J.L., Bartow M.J., Danos D.M., Hilaire H.M.S., Wise M.W., Stalder M.W. (2022). The Impact of Indocyanine Green Angiography on Fat Necrosis in Deep Inferior Epigastric Perforator Flap Breast Reconstruction. Ann. Plast. Surg..

[22] Wapnir I., Dua M., Kieryn A., Paro J., Morrison D., Kahn D., Meyer S., Gurtner G. (2014). Intraoperative imaging of nipple perfusion patterns and ischemic complications in nipple-sparing mastectomies. Ann. Surg. Oncol..

[23] Phillips B.T., Lanier S.T., Conkling N., Wang E.D., Dagum A.B., Ganz J.C., Khan S.U., Bui D.T. (2012). Intraoperative perfusion techniques can accurately predict mastectomy skin flap necrosis in breast reconstruction: Results of a prospective trial. Plast. Reconstr. Surg..

[24] Alstrup T., Christensen B.O., Damsgaard T.E. (2018). ICG angiography in immediate and delayed autologous breast reconstructions: Peroperative evaluation and postoperative outcomes. J. Plast. Surg. Hand Surg..

[25] Gorai K., Inoue K., Saegusa N., Shimamoto R., Takeishi M., Okazaki M., Nakagawa M. (2017). Prediction of Skin Necrosis after Mastectomy for Breast Cancer Using Indocyanine Green Angiography Imaging. Plast. Reconstr. Surg. Glob. Open.

[26] Hammer-Hansen N., Juhl A.A., Damsgaard T.E. (2018). Laser-assisted indocyanine green angiography in implant-based immediate breast reconstruction: A retrospective study. J. Plast. Surg. Hand Surg..

[27] Harless C.A., Jacobson S.R. (2016). Tailoring through Technology: A Retrospective Review of a Single Surgeon’s Experience with Implant-Based Breast Reconstruction before and after Implementation of Laser-Assisted Indocyanine Green Angiography. Breast J..

[28] Mirhaidari S.J., Beddell G.M., Orlando M.V., Parker M.G., Pedersen J.C., Wagner D.S. (2018). A Prospective Study of Immediate Breast Reconstruction with Laser-Assisted Indocyanine Green Angiography. Plast. Reconstr. Surg. Glob. Open..

[29] Rinker B. (2016). A Comparison of Methods to Assess Mastectomy Flap Viability in Skin-Sparing Mastectomy and Immediate Reconstruction: A Prospective Cohort Study. Plast. Reconstr. Surg..

[30] Driessen C., Arnardottir T.H., Lorenzo A.R., Mani M.R. (2020). How should indocyanine green dye angiography be assessed to best predict mastectomy skin flap necrosis? A systematic review. J. Plast. Reconstr. Aesthetic Surg..

[31] Burnier P., Niddam J., Bosc R., Hersant B., Meningaud J.P. (2017). Indocyanine green applications in plastic surgery: A review of the literature. J. Plast. Reconstr. Aesthetic Surg..

[32] Ashok B.C., Kabilan H.K., Anantheswar Y.N., Srikanth V., Somashekar S.P., Prasad A. (2022). Role of Indocyanine Green in Breast Surgery. Indian. J. Surg..

[33] Parmeshwar N., Sultan S.M., Kim E.A., Piper M.L. (2021). A Systematic Review of the Utility of Indocyanine Angiography in Autologous Breast Reconstruction. Ann. Plast. Surg..

[34] Wang Z., Jiao L., Chen S., Li Z., Xiao Y., Du F., Huang J., Long X. (2023). Flap perfusion assessment with indocyanine green angiography in deep inferior epigastric perforator flap breast reconstruction: A systematic review and meta-analysis. Microsurgery.

[35] Steenbeek L.M., Peperkamp K., Ulrich D.J.O., Hummelink S. (2022). Alternative imaging technologies for perforator mapping in free flap breast reconstructive surgery—A comprehensive overview of the current literature. J. Plast. Reconstr. Aesthetic Surg..

[36] Pruimboom T., Schols R.M., Van Kuijk S.M.J., Van der Hulst R.R.W.J., Qiu S.S. (2020). Indocyanine green angiography for preventing postoperative mastectomy skin flap necrosis in immediate breast reconstruction. Cochrane Database Syst. Rev..

[37] Pagliara D., Schiavone L., Garganese G., Bove S., Montella R.A., Costantini M., Rinaldi P.M., Bottosso S., Grieco F., Rubino C. (2023). Predicting Mastectomy Skin Flap Necrosis: A Systematic Review of Preoperative and Intraoperative Assessment Techniques. Clin. Breast Cancer.

[38] Liu E.H., Zhu S.L., Hu J., Wong N., Farrokhyar F., Thoma A. (2019). Intraoperative SPY Reduces Post-mastectomy Skin Flap Complications: A Systematic Review and Meta-Analysis. Plast. Reconstr. Surg—Glob. Open.

[39] Lauritzen E., Damsgaard T.E. (2021). Use of Indocyanine Green Angiography decreases the risk of complications in autologous- and implant-based breast reconstruction: A systematic review and meta-analysis. J. Plast. Reconstr. Aesthetic Surg..

[40] Johnson A.C., Colakoglu S., Chong T.W., Mathes D.W. (2020). Indocyanine green angiography in breast reconstruction: Utility, limitations, and search for standardization. Plast. Reconstr. Surg—Glob. Open.

[41] Shea B.J., Grimshaw J.M., A Wells G., Boers M., Andersson N., Hamel C., Porter A.C., Tugwell P., Moher D., Bouter L.M. (2007). Development of AMSTAR: A measurement tool to assess the methodological quality of systematic reviews. BMC Med. Res. Methodol..

[42] Lie K.H., Barker A.S., Ashton M.W. (2013). A classification system for partial and complete diep flap necrosis based on a review of 17,096 DIEP flaps in 693 articles including analysis of 152 total flap failures. Plast. Reconstr. Surg..

[43] Ogawa A., Nakagawa T., Oda G., Hosoya T., Hayashi K., Yoshino M., Mori H., Uemura N., Fujioka T., Mori M. (2021). Study of the protocol used to evaluate skin-flap perfusion in mastectomy based on the characteristics of indocyanine green. Photodiagnosis Photodyn. Ther..

[44] Wagner I.J., Tong W.M., Halvorson E.G. (2013). A classification system for fat necrosis in autologous breast reconstruction. Ann. Plast. Surg..

[45] Oleck N.C., Gu C., Pyfer B.J., Phillips B.T. (2022). Defining Mastectomy Skin Flap Necrosis: A Systematic Review of the Literature and a Call for Standardization. Plast. Reconstr. Surg..

[46] Jong L.J.S., Appelman J.G.C., Sterenborg H.J.C.M., Ruers T.J.M., Dashtbozorg B. (2024). Spatial and Spectral Reconstruction of Breast Lumpectomy Hyperspectral Images. Sensors.

[47] Thiem D.G.E., Frick R.W., Goetze E., Gielisch M., Al-Nawas B., Kämmerer P.W. (2021). Hyperspectral analysis for perioperative perfusion monitoring—A clinical feasibility study on free and pedicled flaps. Clin. Oral. Investig..

[48] Pruimboom T., Lindelauf A.A.M.A., Felli E., Sawor J.H., Deliaert A.E.K., van der Hulst R.R.W.J., Al-Taher M., Diana M., Schols R.M. (2022). Perioperative Hyperspectral Imaging to Assess Mastectomy Skin Flap and DIEP Flap Perfusion in Immediate Autologous Breast Reconstruction: A Pilot Study. Diagnostics.

[49] Shapey J., Xie Y., Nabavi E., Bradford R., Saeed S.R., Ourselin S., Vercauteren T. (2019). Intraoperative multispectral and hyperspectral label-free imaging: A systematic review of in vivo clinical studies. J. Biophotonics.

[50] Zötterman J., Bergkvist M., Iredahl F., Tesselaar E., Farnebo S. (2016). Monitoring of partial and full venous outflow obstruction in a porcine flap model using laser speckle contrast imaging. J. Plast. Reconstr. Aesthetic Surg..

[51] Zötterman J., Opsomer D., Farnebo S., Blondeel P., Monstrey S., Tesselaar E. (2020). Intraoperative Laser Speckle Contrast Imaging in DIEP Breast Reconstruction: A Prospective Case Series Study. Plast. Reconstr. Surg—Glob. Open.

[52] Zötterman J., Tesselaar E., Farnebo S. (2019). The use of laser speckle contrast imaging to predict flap necrosis: An experimental study in a porcine flap model. J. Plast. Reconstr. Aesthetic Surg..

[53] Zötterman J., Tesselaar E., Elawa S., Farnebo S. (2023). Correlation between Indocyanine Green Fluorescence Angiography and Laser Speckle Contrast Imaging in a Flap Model. Plast. Reconstr. Surg—Glob. Open.

[54] Attia A.B.E., Balasundaram G., Moothanchery M., Dinish U., Bi R., Ntziachristos V., Olivo M. (2019). A review of clinical photoacoustic imaging: Current and future trends. Photoacoustics.

[55] Zhang D., Chen H., Hu X., Yu A. (2021). Photoacoustic microscopy: A novel approach for studying perforator skin flap in a mouse model. Quant. Imaging Med. Surg..

